# Novel Ozark Orthohantavirus in Hispid Cotton Rats (*Sigmodon hispidus*), Arkansas, USA

**DOI:** 10.3201/eid2912.230549

**Published:** 2023-12

**Authors:** Nathaniel Mull, Mert Erdin, Teemu Smura, Tarja Sironen, Kristian M. Forbes

**Affiliations:** University of Arkansas, Fayetteville, Arkansas, USA (N. Mull, K.M. Forbes);; University of Helsinki, Helsinki, Finland (M. Erdin, T. Smura, T. Sironen)

**Keywords:** orthohantavirus, hantavirus, hantavirus pulmonary syndrome, phylogeny, prevalence, sequence analysis, respiratory infections, cotton rats, viruses, zoonoses, Arkansas, United States

## Abstract

We report a novel orthohantavirus, putatively named Ozark orthohantavirus, in hispid cotton rats captured within the Ozark Plateau in Arkansas, USA. This virus phylogenetically clusters with other orthohantaviruses that cause severe human disease. Continued orthohantavirus surveillance and virus sequencing are needed to address the potential public health threat of this virus.

Orthohantaviruses (family Hantaviridae, genus *Orthohantavirus*) are a group of zoonotic viruses primarily found in muroid rodents; many of the viruses are pathogenic in humans (*1*). Pathogenic orthohantaviruses in the Americas are hosted by rodents in subfamilies Sigmodontinae and Neotominae and cause hantavirus cardiopulmonary syndrome (HCPS) in humans, which has a 30%–40% case-fatality rate (*2*,*3*). Although several pathogenic orthohantaviruses have been identified in the Americas, the specific etiologic virus is unknown for many HCPS cases (*2*).

We report a novel orthohantavirus species, putatively named Ozark orthohantavirus or Ozark virus (OZV), in hispid cotton rats (*Sigmodon hispidus*) in Arkansas, USA. Hispid cotton rats are a reservoir host of a notable pathogenic orthohantavirus, Black Creek Canal virus (BCCV) (*4*), in the United States and have also been identified as the host of the proposed Muleshoe virus (*5*). Despite the wide distribution of hispid cotton rats in North America (22 US states and northern Mexico), previously published orthohantavirus surveillance and detection in this rat species has been limited to only Florida and Texas in the United States.

We analyzed frozen lung tissue samples collected from euthanized hispid cotton rats previously captured during 2020 and 2021 in the Ozark Plateau region of Arkansas, USA (*6*). Of 338 rat samples previously tested, 26 (7.7%) were orthohantavirus-seropositive; seropositive rats had been captured in 5 distinct grassland sites (*6*).

We performed homogenization, filtration, and nuclease pretreatment of available lung tissue samples from 13 orthohantavirus-seropositive rodents captured in 3 of the 5 unique grassland sites (Appendix Table) (*7*,*8*). We then extracted RNA by using Invitrogen TRIzol (Thermo Fisher Scientific, https://www.thermofisher.com) according to manufacturer guidelines. We used the NEBNext rRNA Depletion Kit (human/mouse/rat) to remove host rRNA, then the NEBNext Ultra II RNA Library Prep Kit (both from New England Biolabs, https://www.neb.com) to construct libraries. We performed next-generation sequencing by using the Illumina NovaSeq system (https://www.illumina.com). We quality filtered and de novo assembled the raw data and annotated the contigs by using LazyPipe (*9*).

We obtained complete genome sequences of OZV coding regions for small (S), medium (M), and large (L) segments from 2 rat samples and partial genome sequences from 6 other rat samples that included 3 additional complete S and 4 additional complete M segment sequences (Appendix Table). We used Open Reading Frame (ORF) Finder (https://www.ncbi.nlm.nih.gov/orffinder) to detect ORFs and the Expasy translate tool (https://www.expasy.org) to translate ORFs to amino acid sequences. We compared corresponding nucleic acid and protein phylogenies of each OZV genome segment with BCCV and other related orthohantavirus sequences obtained from GenBank by using IQ-TREE2 (http://www.iqtree.org). We then used the Sequence Demarcation Tool version 1.2 program (http://web.cbio.uct.ac.za/~brejnev) to compare protein sequence pairwise identities of each OZV segment with those of closely related orthohantaviruses. Finally, we performed pairwise evolutionary distance (PED) analyses by using TREE-PUZZLE version 5.2 (http://www.tree-puzzle.de) with a PED cutoff value of 0.1 for species classification (*10*).

OZV nucleotide sequences most closely clustered with other sigmodontine-borne orthohantaviruses, particularly BCCV and Bayou virus (BAYV), which are pathogenic to humans, and Catacamas virus (CATV), which is not known to cause human infections (*2*). OZV S segment contig lengths were 1,988 and 1,884 nt and were 80.84% similar to BCCV, 81.15% similar to BAYV, and 80.93% similar to CATV S gene segments (Appendix Figures 1). OZV M segment contig lengths were 3,690 and 3,709 nt and were 77.91% similar to BCCV and 78.11% similar to BAYV (Appendix Figures 2). OZV L segment contig lengths were 6,523 and 6,462 nt and were 80.32% similar to BCCV, 80.16% similar to BAYV, and 80.01% similar to CATV (Appendix Figures 3). Pairwise relationships for protein sequences among OZV and related viruses were similar to those observed for nucleotide sequences (Figure; Appendix Figures 4–6). PED results for sigmodontine- and neotomine-borne orthohantaviruses indicated that OZV is a novel species with a PED value >0.1 and is closely related to BCCV, BAYV, and CATV (Appendix Figure 7).

**Figure:**
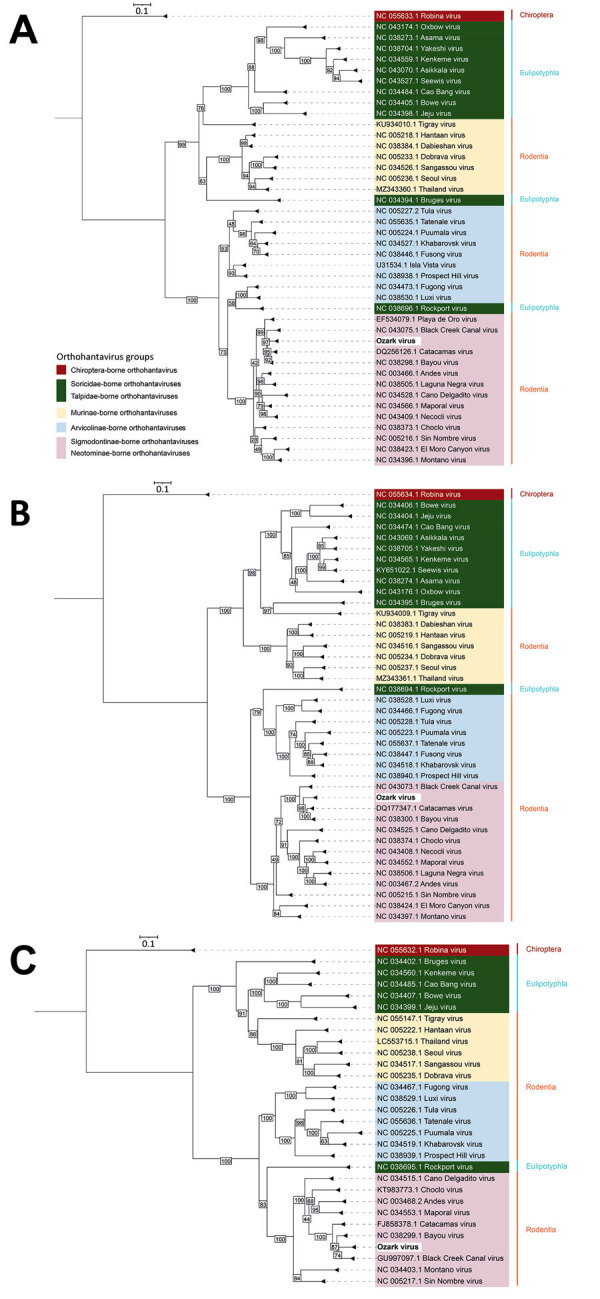
Phylogenetic analysis of novel Ozark orthohantavirus segments isolated from hispid cotton rats (*Sigmodon* *hispidus*), Arkansas, USA. Phylogenetic trees were constructed by using IQ-TREE2 (http://www.iqtree.org) for small (A), medium (B), and large (C) protein segments translated from Ozark orthohantavirus open reading frames (ORFs). ORF Finder (https://www.ncbi.nlm.nih.gov/orffinder) was used to detect ORFs and the Expasy translate tool (https://www.expasy.org) was used to translate ORFs to amino acid sequences. Maximum-likelihood method and best-fit models Q matrix estimated for insects (Q.insect) plus proportion of invariable sites enabled plus discreet gamma model added with 4 rate categories (for small segment), Q.insect plus FreeRate model with 5 categories (for medium segment), and Q.insect plus proportion of invariable sites enabled plus invariable sites plus FreeRate model with 4 categories (for large segment) were used (http://www.iqtree.org/doc/Substitution-Models). Sequences and corresponding GenBank accession numbers are indicated for available orthohantaviruses from orders Chiroptera (bats), Eulipotyphla, and Rodentia. Orthohantaviruses from Eulipotyphla are found in families Soricidae (shrews) and Talpidae (moles); orthohantaviruses from Rodentia are found in family Muridae, subfamily Murinae (Old World mice and rats) and family Cricetidae, subfamilies Arvicolinae (voles and lemmings) and Sigmodontinae and Neotominae (both New World mice and rats). Hispid cotton rats are sigmodontine rodents. Scale bar indicates amino acid substitutions per site.

OZV is the second definitive orthohantavirus species identified in hispid cotton rats. This discovery also expands the geographic distribution of orthohantavirus-carrying hispid cotton rats in the United States, previously limited to Florida and Texas; because of OZV’s similarity to BCCV and BAYV, which cause severe disease, this discovery provides crucial public health information. OZV identification also informs broader orthohantavirus evolution, especially for within-host evolution and divergence. Although uncommon, multiple orthohantaviruses in a single reservoir host species have been observed, particularly in cricetid-borne orthohantaviruses in the Americas (*3*).

In conclusion, hispid cotton rats are primarily found in grassland and agricultural habitats, and their range comprises the entire state of Arkansas. At least 1 HCPS case has been recorded in Arkansas; because of its close phylogenetic relationship with known human pathogens, OZV should be considered a potential cause of future HCPS cases in Arkansas, surrounding states, and other areas that harbor hispid cotton rats. Continued surveillance is needed to address the potential public health threat of OZV throughout the distribution range of the hispid cotton rat host.

Appendix Additional information for novel Ozark orthohantavirus in hispid cotton rats (*Sigmodon* *hispidus*), Arkansas, USA.
