# Supplementary material for: Novel Ozark Orthohantavirus in Hispid Cotton Rats (Sigmodon hispidus), Arkansas, USA
Source: Emerg Infect Dis. 2023 Dec;29(12):2576–8. doi: 10.3201/eid2912.230549 (PMC10683798; doi:10.3201/eid2912.230549)
Supplement: Appendix — Additional information for novel Ozark orthohantavirus in hispid cotton rats (Sigmodon hispidus), Arkansas, USA. [file 23-0549-Techapp-s1.pdf]

Article DOI: https://doi.org/10.3201/eid2912.230549

EID cannot ensure accessibility for supplementary materials supplied by authors. Readers who have difficulty accessing supplementary content should contact the authors for assistance.

Novel Ozark Orthohantavirus in Hispid Cotton Rats (*Sigmodon hispidus*), Arkansas, USA

Appendix

Appendix Table. Hispid cotton rat characteristics and sequencing results for small, medium, and large gene segments of Ozark virus isolated from rat lung tissue samples*

Animal ID	Small segment	Medium segment	Large segment	Trapping site†	Sex	Rat weight, g
8	P	C	P	STUMP	F	126
12	N	N	N	WOOL_B	M	146
45	N	N	N	STUMP	M	140
208	C	C	P	CHES_C	M	129
217	C	C	P	CHES_C	M	207
218	N	N	N	CHES_C	F	133
220	C	C	C	CHES_C	M	153
227	C	C	C	STUMP	M	139
304	P	P	P	STUMP	M	137
315	N	N	N	WOOL_B	F	81
322	C	C	P	STUMP	M	237
325	P	P	P	STUMP	M	196
332	N	N	N	CHES_C	M	69

*More details on trapping site locations and hispid cotton rat measurements are presented in (*6*, main text). C, complete sequence; CHES_C, Chesney Prairie Natural Area, area C; N, no sequence results; P, partial sequence; STUMP, Stump's Prairie; WOOL_B, Woolsey Wet Prairie, area B.

†Trapping sites were in Benton and Washington Counties, Arkansas, USA.

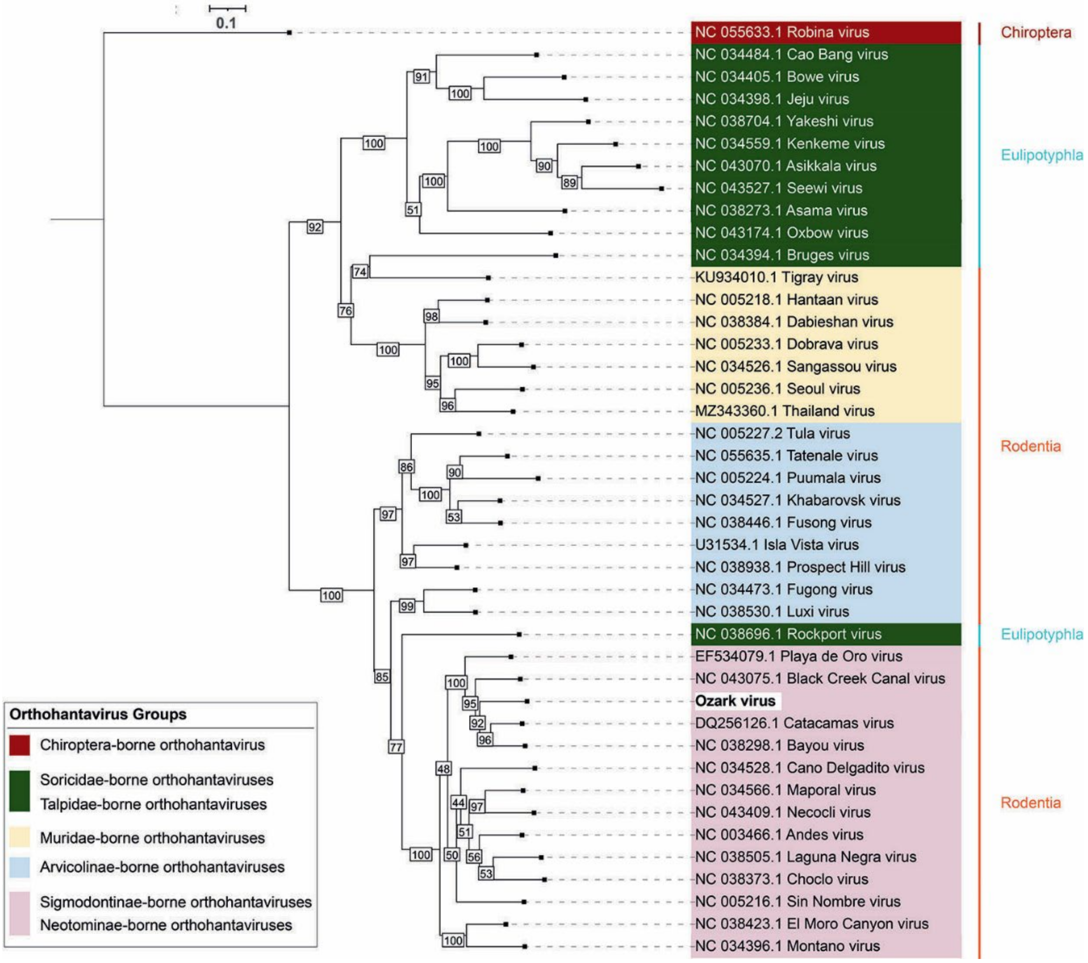

Appendix Figure 1. Phylogenetic analysis of the small gene segment from Ozark virus and other orthohantaviruses. Maximum-likelihood phylogenetic tree of open reading frames of orthohantavirus small gene segments was constructed by using IQ-TREE2 (http://www.iqtree.org) and the best-fit model: general time reversible + empirical base frequencies + proportion of invariable sites enabled + invariable sites + FreeRate model with five categories. Orthohantavirus sequences and corresponding accession numbers were retrieved from GenBank. Ozark virus found in hispid cotton rats, which are sigmodontine rodents, is highlighted in white. Scale bar indicates nucleotide substitutions per site.

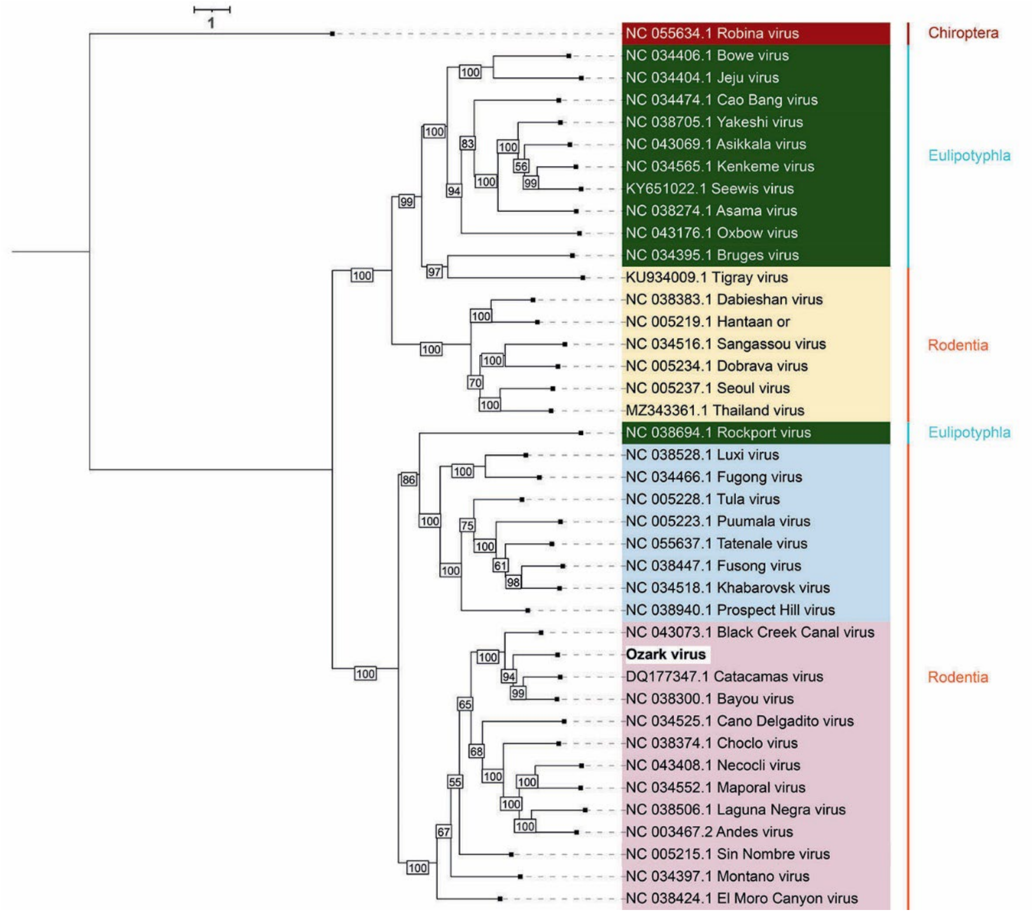

Appendix Figure 2. Phylogenetic analysis of medium gene segment from Ozark virus and other orthohantaviruses. Maximum-likelihood phylogenetic tree of open reading frames of orthohantavirus medium gene segments was constructed by using IQ-TREE2 (http://www.iqtree.org) and the best-fit model: general time reversible + empirical base frequencies + FreeRate model with six categories. Orthohantavirus sequences and corresponding accession numbers were retrieved from GenBank. Ozark virus found in hispid cotton rats, which are sigmodontine rodents, is highlighted in white. Scale bar indicates nucleotide substitutions per site.

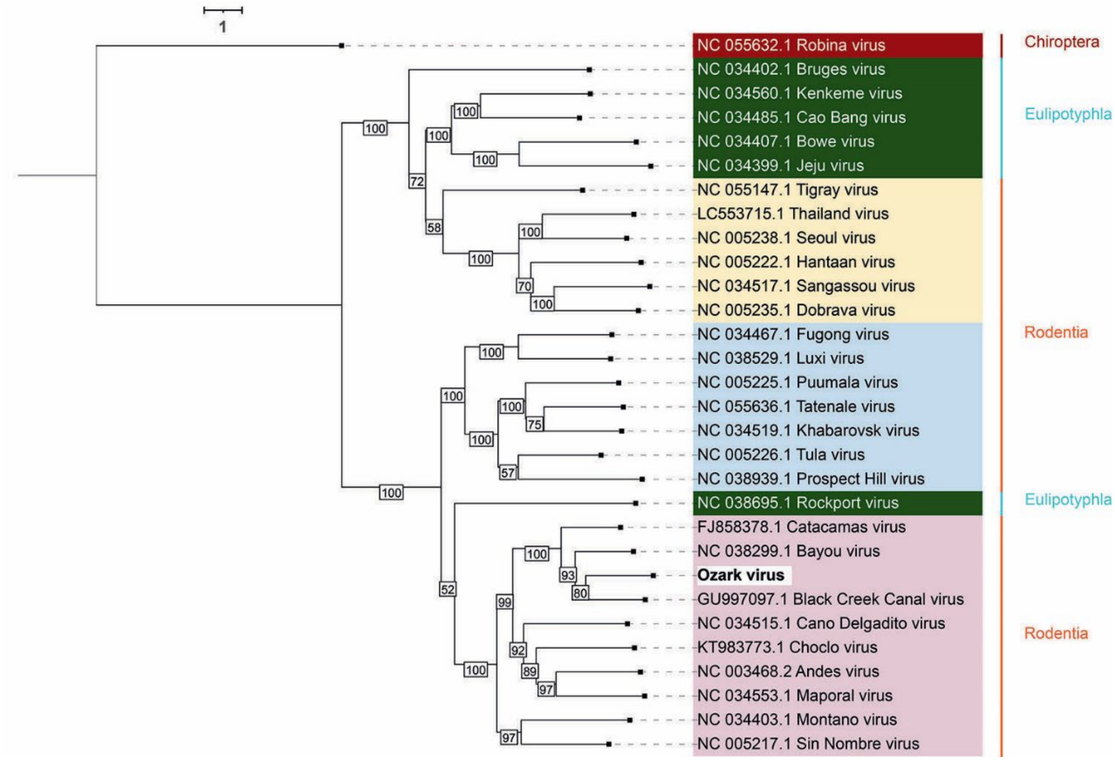

Appendix Figure 3. Phylogenetic analysis of large gene segment from Ozark virus and other orthohantaviruses. Maximum-likelihood phylogenetic tree of open reading frames of orthohantavirus large gene segments was constructed by using IQ-TREE2 (http://www.iqtree.org) and the best-fit model: general time reversible + empirical base frequencies + proportion of invariable sites enabled + invariable sites and FreeRate model with five categories. Orthohantavirus sequences and corresponding accession numbers were retrieved from GenBank. Ozark virus found in hispid cotton rats, which are sigmodontine rodents, is highlighted in white. Scale bar indicates nucleotide substitutions per site.

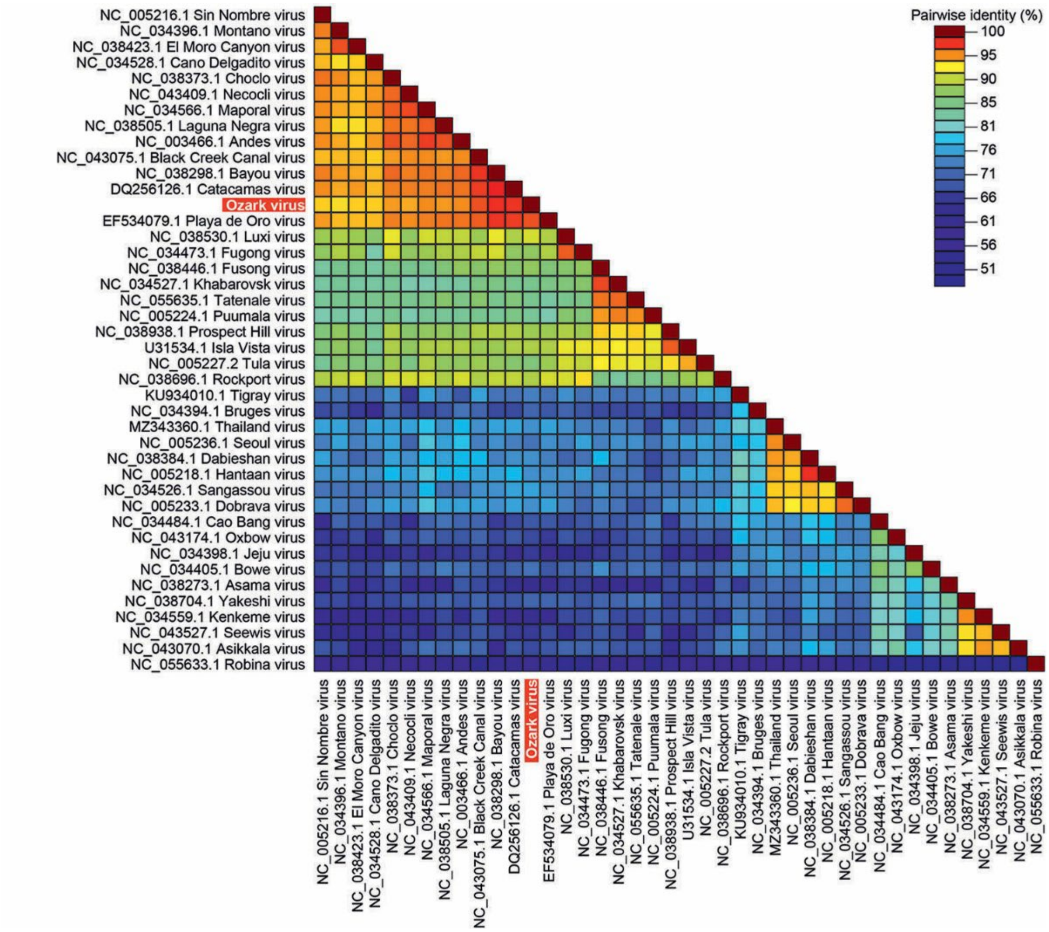

Appendix Figure 4. Pairwise comparisons of small protein segments translated from open reading frames (ORFs) of Ozark virus and other orthohantaviruses. ORF Finder (https://www.ncbi.nlm.nih.gov/orffinder) was used to detect ORFs and the Expasy translate tool (https://www.expasy.org) was used to translate ORFs to amino acid sequences. Sequence Demarcation Tool (http://web.cbio.uct.ac.za/~brejnev) was used to compare pairwise identities of small protein segments. Colors indicate different percentages of pairwise identity between viruses.

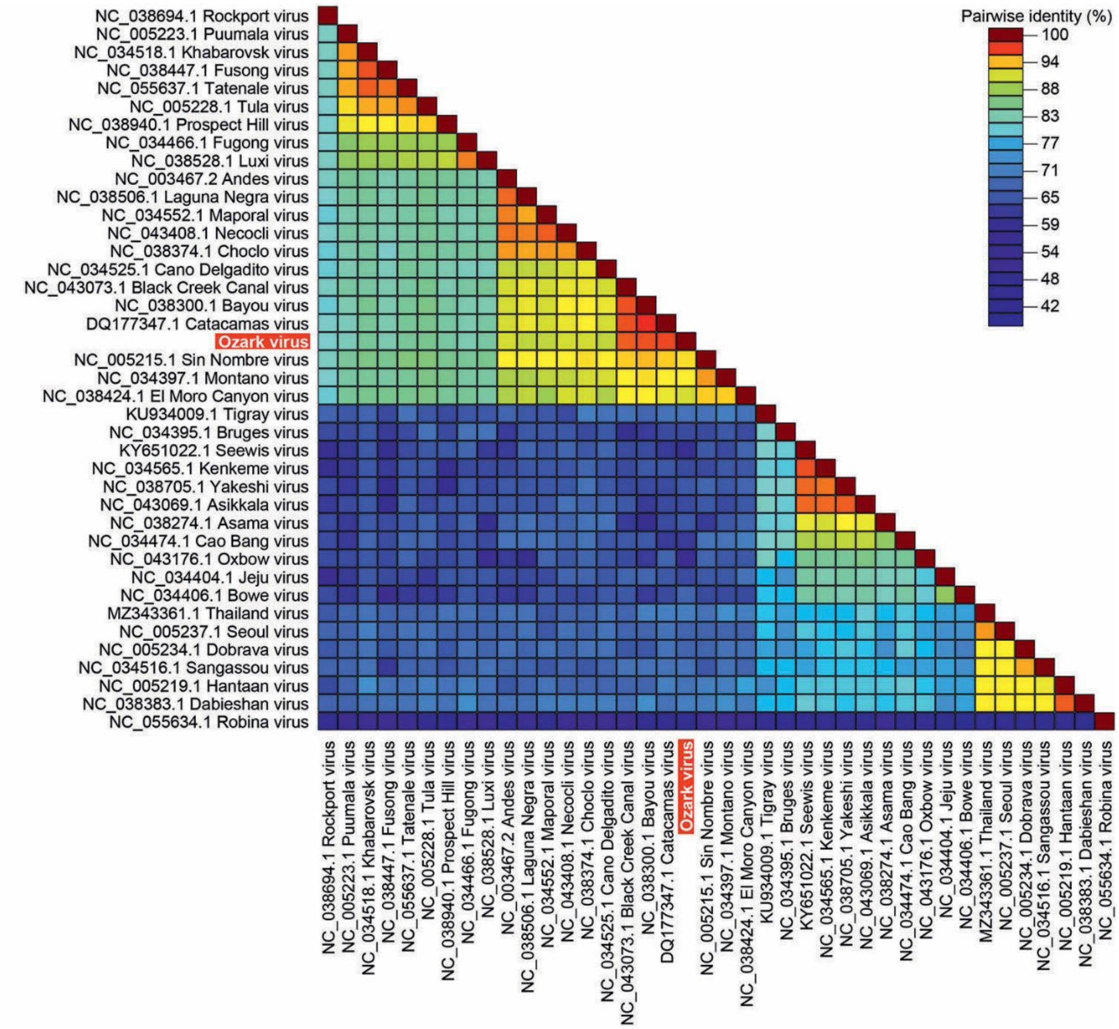

Appendix Figure 5. Pairwise comparisons of medium protein segments translated from open reading frames (ORFs) of Ozark virus and other orthohantaviruses. ORF Finder (https://www.ncbi.nlm.nih.gov/orffinder) was used to detect ORFs and the Expasy translate tool (https://www.expasy.org) was used to translate ORFs to amino acid sequences. Sequence Demarcation Tool (http://web.cbio.uct.ac.za/~brejnev) was used to compare pairwise identities of medium protein segments. Colors indicate different percentages of pairwise identity between viruses.

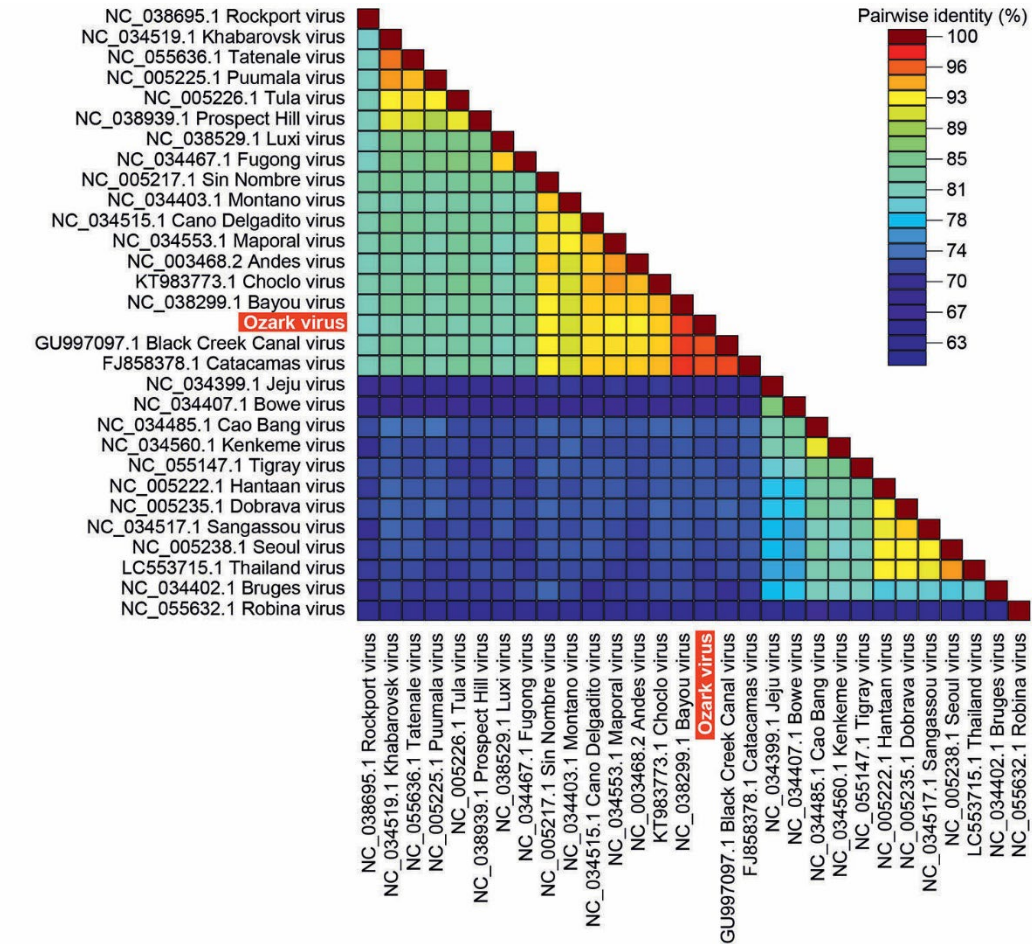

Appendix Figure 6. Pairwise comparisons of large protein segments translated from open reading frames (ORFs) of Ozark virus and other orthohantaviruses. ORF Finder (https://www.ncbi.nlm.nih.gov/orffinder) was used to detect ORFs and the Expasy translate tool (https://www.expasy.org) was used to translate ORFs to amino acid sequences. Sequence Demarcation Tool (http://web.cbio.uct.ac.za/~brejnev) was used to compare pairwise identities of large protein segments. Colors indicate different percentages of pairwise identity between viruses.

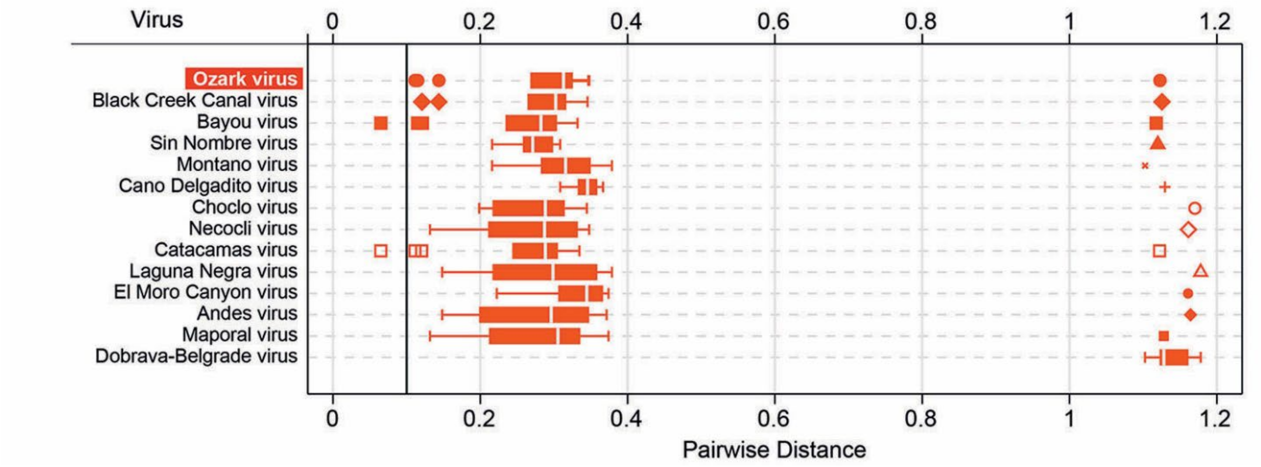

Appendix Figure 7. Pairwise evolutionary distance analyses of Ozark virus compared with other orthohantaviruses. TREE-PUZZLE (http://www.tree-puzzle.de) was used to determine pairwise evolutionary distance; cutoff value was 0.1 for species recognition. Higher pairwise distance values reflect greater pairwise divergence between viruses. Bars indicate the mean distributional range of divergence values; longer bars indicate greater variation in divergence values. Circle, squares, and triangles represent outliers for each virus indicating single divergence values.
